# Testing Protocols and Procedures for Undertaking Fire Resistance Tests on Concrete Structures Incorporating Fiber-Reinforced Polymers

**DOI:** 10.3390/polym17030404

**Published:** 2025-02-03

**Authors:** Venkatesh Kodur, M. Z. Naser, Hee Sun Kim

**Affiliations:** 1 Department of Civil and Environmental Engineering, Michigan State University, East Lansing, MI 48824, USA; 2 Department of Architectural and Urban Systems Engineering, Ewha Womans University, Seoul 03760, Republic of Korea; hskim3@ewha.ac.kr; 3 School of Civil and Environmental Engineering & Earth Sciences, Clemson University, Clemson, SC 29634, USA; mznaser@clemson.edu

**Keywords:** fiber reinforced polymers, fire resistance, fire testing protocols, concrete structures, non-metallic reinforcement, strengthening and retrofitting

## Abstract

Fiber-reinforced polymers (FRPs) are often incorporated as internal (primary) reinforcement in new concrete constructions or as external (secondary) reinforcement in retrofitting and strengthening of existing concrete structures. Under fire conditions, the response of FRP-incorporated concrete structures are altered due to the presence of FRPs; thus, their fire performance is different from that of concrete structures with conventional metallic reinforcement. However, the fire resistance of these FRP-incorporated structural members continues to be evaluated through standard fire resistance tests, which are similar to conventional steel and concrete structural members. Despite the complexity of this testing approach and its drawbacks, standard fire testing remains a cornerstone in evaluating FRP-incorporated concrete structural members. Thus, this paper sheds more light on the fire testing procedure and discusses the distinctive factors that differentiate the fire performance of FRP-incorporated concrete structures from that of conventional concrete structures and the need for additional provisions to test such structures. To address the current shortcomings, a set of additional testing protocols and procedures for undertaking fire resistance tests on FRP-incorporated concrete structural members are presented. The performance criteria to be applied to evaluate the failure of FRP–RC structural members under fire conditions are discussed.

## 1. Introduction

Fiber Reinforced Polymer (FRP) reinforcement has recently been recognized as an effective alternative to metallic reinforcement in concrete structures. This is mainly due to the numerous advantages of the FRP over steel, which include a high strength-to-weight ratio, good corrosion resistance, and ease of application. FRPs are composite materials consisting of a polymer matrix (typically epoxy, polyester, or vinyl ester) reinforced with fibers, typically carbon (CFRP), glass (GFRP), basalt (BFRP or aramid (AFRP), each with unique properties and applications.

**Figure 1:**
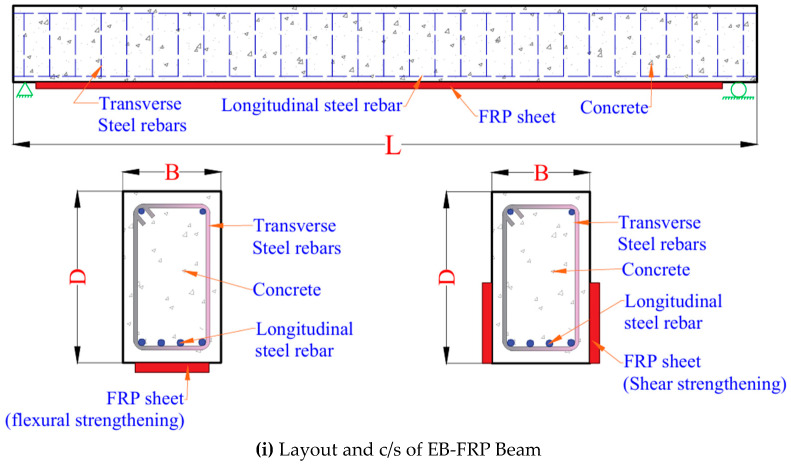

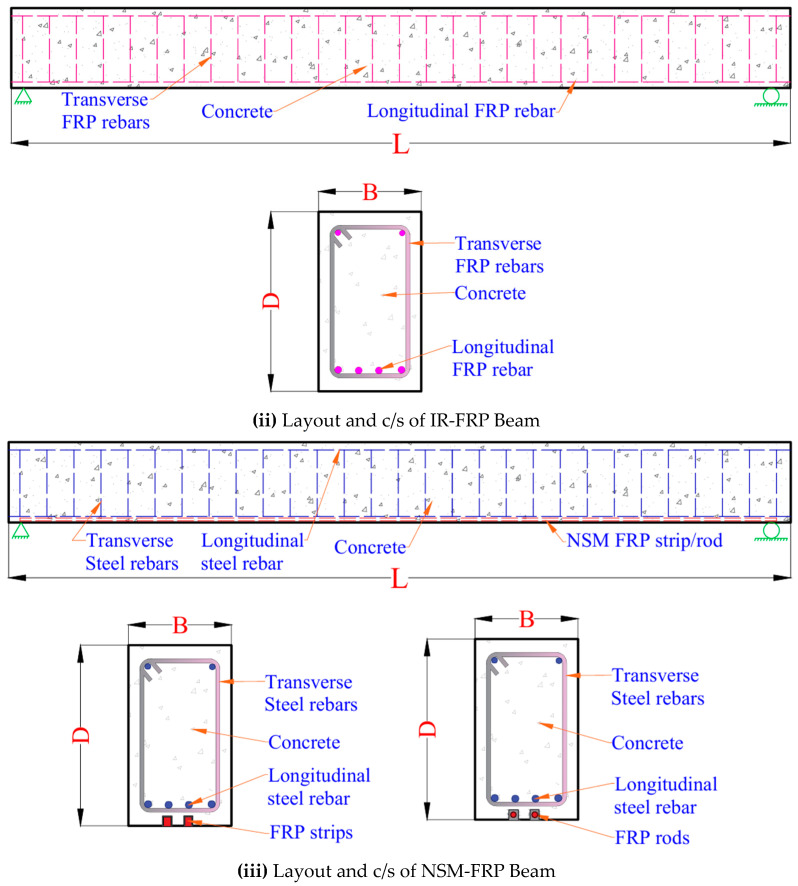
Applications for the use of FRP in reinforced concrete structures (IR, EB, and NSM).

FRP reinforcement is typically incorporated in concrete structures through one of three configurations, namely, internally reinforced (IR), externally bonded (EB), or near-surface mounted (NSM) technique [1]. The typical applications for FRP in RC beams (such as IR, EB, and NSM) (see Figure 1). Similarly, FRP can be incorporated into other concrete members such as columns, slabs, walls, or chimneys. In the case of new construction, the IR technique is adopted wherein FRP is applied as primary internal reinforcement, instead of traditional steel reinforcement, in beams, columns, slabs, or walls, and this can overcome the problem of corrosion and durability concerns in concrete structures.

FRP is also used as a secondary reinforcement to strengthen and rehabilitate existing concrete structures. Two techniques can be employed for deploying FRP in such cases: externally bonded (EB) FRP reinforcement or Near Surface Mounted (NSM) internal reinforcement. In EB strengthening, FRP plates, strips, or sheets are applied to the surface of a concrete member after preparing the concrete external surface through grinding, sandblasting, or high-pressure water jet to remove the weak concrete surfaces. In the case of columns or walls, FRP sheets are wrapped around the exterior sides of the column or wall. In the case of flexural members (beams or slabs), the FRP plates or strips are attached to the soffit (and sides) of the beam/slab. Applying FRP through the EB strengthening technique is direct and involves less effort and time. However, one of the major drawbacks is that the externally applied FRP cannot develop full bonding with the concrete surface, as only one side of the FRP (attachment) is in contact with the concrete surface. Also, low levels of axial strain develop in FRP. Thus, in the EB strengthening method, FRP’s full tensile strength potential cannot effectively be utilized [2]. In order to overcome such limitations, an alternative NSM FRP strengthening technique has recently emerged [3].

The NSM technique involves the installation of FRP reinforcement elements, either in strip or rod configuration, within preformed channels excavated in the concrete cover of RC structural elements. These cavities are subsequently filled with structural binding agents, specifically epoxy-based adhesives or cementitious grouting compounds. When introduced into the prepared cavity, the binding medium facilitates optimal mechanical anchorage between the NSM FRP element and the concrete substrate. This enables the composite system to function as supplementary tension reinforcement under applied loads. In comparison to EB strengthening systems, NSM applications demonstrate superior interfacial bond characteristics due to the multi-directional adhesion surface created between the reinforcement element and the binding medium [3].

In construction applications, structural concrete members incorporating FRP must comply with fire resistance specifications mandated by building regulations [4]. While traditionally RC elements with steel reinforcement typically meet prescribed fire resistance criteria without supplementary fire protection measures, the integration of internal or external FRP composites significantly alters the thermal response characteristics of these structural components [5,6]. The recent integration of FRP in building elements has raised critical concerns regarding their performance under fire conditions, primarily due to FRP’s inherent combustibility and pronounced degradation of mechanical properties and bond strength when subjected to elevated temperatures [7,8]. These vulnerability factors represent substantial impediments to the widespread adoption of FRP in reinforced concrete structures, particularly in building applications where fire safety considerations constitute fundamental design criteria.

An increasing number of fire tests and numerical studies have been conducted to understand the behavior of FRP-incorporated RC members over the past two decades [6,9,10,11]. However, fire resistance tests on these FRP-incorporated structural members have been mostly conducted through standard fire resistance tests, as applied to conventional steel and concrete structural members. This standard fire testing approach has numerous drawbacks and may not yield realistic fire performance in FRP-incorporated structural members. From this lens, this paper discusses the key factors that distinguish FRP-incorporated concrete structures’ fire performance from conventional concrete structures and the need to incorporate additional provisions for undertaking fire testing of such structures. Then, new testing protocols and procedures are presented, including performance criteria for evaluating failure and undertaking fire resistance tests on FRP-incorporated concrete structural members.

## 2. The Need for Fire Resistance in FRP–RC Structures

Fire hazards pose a critical threat to built infrastructure and necessitate the implementation of codified fire resistance requirements for structural elements [4]. These regulatory specifications mandate fire resistance ratings ranging from 1 to 4 h, with specific requirements determined by building classification parameters, including occupancy category, vertical height, and floor area dimensions. Standard reinforced or prestressed concrete elements typically achieve prescribed fire resistance ratings inherently, contingent upon maintaining specified concrete cover over steel reinforcement or tendons. However, the incorporation of FRP reinforcement in concrete elements can result in diminished fire resistance characteristics (specifically, reduced load-bearing capacity under thermal loading) compared to equivalent steel-reinforced configurations. This performance differential stems from the fire resistance being governed by both concrete properties and FRP material characteristics, with FRP exhibiting more severe degradation of mechanical properties than steel or concrete at elevated temperatures [12,13]. Furthermore, FRP’s inherent flammability and accelerated deterioration of bond properties under heating result in expedited reduction in cross sectional capacities compared to conventional RC elements [14].

## 3. Fire Performance of FRP-Incorporated Concrete Structures

Poor performance of FRP-incorporated concrete structures under fire exposure is limiting the wider use of FRP in building applications. This poor fire performance mainly results from the rapid deterioration of FRP composites’ properties and a lack of understanding of the realistic behavior of FRP-incorporated RC members under fire conditions.

### 3.1. Performance Issues with FRP Under Elevated Temperature Exposure

FRP composites often consist of fiber reinforcement and polymer matrices and exhibit excellent characteristics under ambient conditions. However, thermal exposure induces polymer matrix decomposition, resulting in the emission of heat, smoke and carbonaceous particles, etc. From a structural mechanics perspective, the composite system experiences rapid degradation of mechanical properties at elevated temperatures. Furthermore, in EB and NSM applications where FRP is adhered to concrete substrates using epoxy-based systems, the interfacial bond properties deteriorate substantially when the FRP temperature exceeds the glass transition temperature (T_g_) of the epoxy matrix, compromising the load transfer mechanism between the concrete substrate and FRP reinforcement.

While epoxy-based adhesive systems exhibit satisfactory bond characteristics at ambient temperatures, these systems undergo significant softening at relatively low temperatures (approximately 65 °C) and results in marked deterioration of FRP-concrete bond strength beyond T_g_. When the interfacial shear stress exceeds the temperature-degraded bond strength at T_g_, delamination of FRP laminates occurs [14]. Post-debonding, the FRP reinforcement’s contribution to the structural member’s load-carrying capacity becomes negligible across all mechanical response modes.

**Figure 2:**
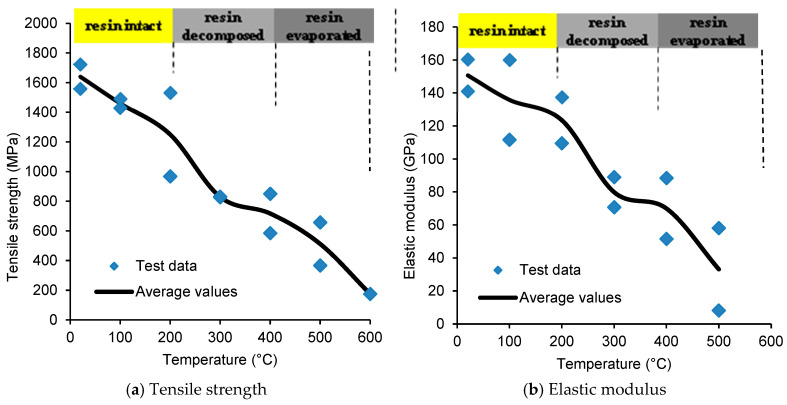
Degradation of tensile strength and elastic modulus of CFRP strips with temperature.

Beyond interfacial degradation, FRP’s mechanical properties, specifically tensile strength and elastic modulus, experience rapid deterioration at elevated temperatures due to polymer matrix phase transition. Figure 2a illustrates the thermal degradation profiles of CFRP and GFRP mechanical properties across the 20–1000 °C range. CFRP and GFRP reinforcement exhibit 50% strength retention at approximately 325 °C and 250 °C, respectively [12], defining their critical temperatures—analogous to steel reinforcement’s critical temperature of 593 °C. Post-critical temperature exposure results in extensive polymer matrix decomposition, though residual fiber reinforcement maintains a limited contribution to moment capacity [14,15]. Given the limited empirical data on post-decomposition mechanical properties, conventional modeling approaches assume linear strength and modulus degradation from 40% (CFRP) or 25% (GFRP) to complete deterioration across 400–1000 °C, with total strength loss assumed at 1000 °C [12].

In addition to tensile strength, the development of an effective bond between FRP and concrete is critical for transferring stresses from the FRP reinforcement to the concrete and vice versa [10,16]. Such load transfer occurs through shear stresses that develop in the polymer matrix or adhesive layer. Due to temperature-induced changes in the state and composition of the matrix material (resin), the bond property of FRP is affected even by a moderate rise in temperature (as low as 80 °C). Thus, a rise in temperature in FRP leads to a loss of interaction (bond) between FRP and the concrete surface, which in turn reduces the load-carrying capacity of an FRP-incorporated concrete member.

**Figure 3:**
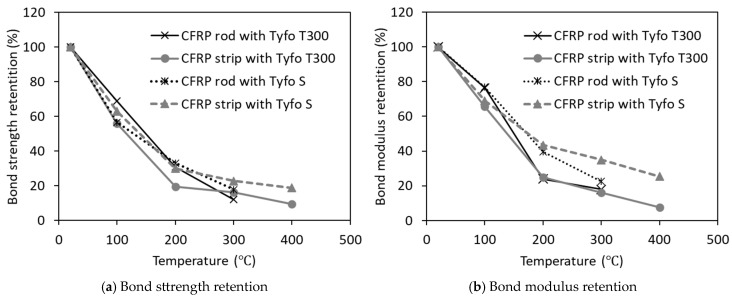
Degradation of bond strength and bond modulus of CFRP strips with temperature.

The thermal dependence of bond strength and bond modulus characteristics across various FRP typologies, extending to 400 °C can be seen in Figure 3. This figure shows observations derived from experimental investigations conducted by Yu and Kodur [14]. Both CFRP rebar variants exhibit pronounced degradation of bond strength and modulus parameters upon exceeding their respective glass transition temperatures. The bond strength approaches negligible values at approximately 400 °C, at which point interfacial slip mechanisms may initiate additional degradation to structural capacity. Comparative analysis of Figure 2 and Figure 3 reveals that bond strength degradation in CFRP systems progresses at a markedly accelerated rate relative to tensile strength reduction. GFRP reinforcement systems in bar or laminate configurations demonstrate analogous bond degradation patterns to their CFRP counterparts.

### 3.2. Response of FRP-Incorporated Concrete Structure to Fire

The properties of constituent materials influence the fire response of a structural member, and the failure occurs when the capacity (resulting from the strength contribution of all constituent materials) of the member falls below the load effects present during fire exposure. Typically, a fire-exposed conventional concrete member with metallic reinforcement experiences gradual degradation in capacity, mainly resulting from the temperature-induced strength loss in steel rebars. Thus, it can achieve the required fire resistance with the provision of sufficient concrete cover for steel rebars. However, in the case of FRP-incorporated RC members, FRP reinforcement undergoes faster strength and bond degradation with temperature than steel rebar (if any) and concrete. Further, when the glass transition temperature of FRP (T_g_) is reached, which is around 80 °C for epoxy-based adhesives, the matrix softens, and thus, the bond (strength) between FRPs and concrete decreases significantly. For these reasons, an FRP-reinforced member’s capacity decreases faster in the early stages of fire exposure. Once the full effectiveness of FRP to strength contribution is gone, the capacity degradation is mainly controlled by the properties of concrete and steel reinforcement (if any), and thus, the behavior of the FRP–RC member at later stages of fire exposure follows that of an RC member [13].

**Figure 4:**
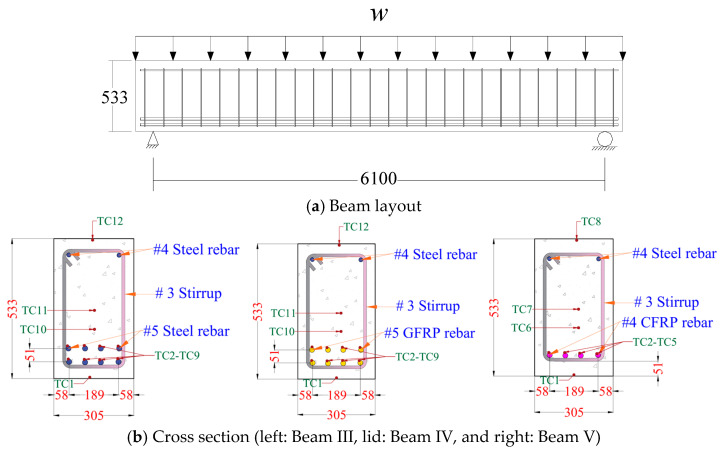

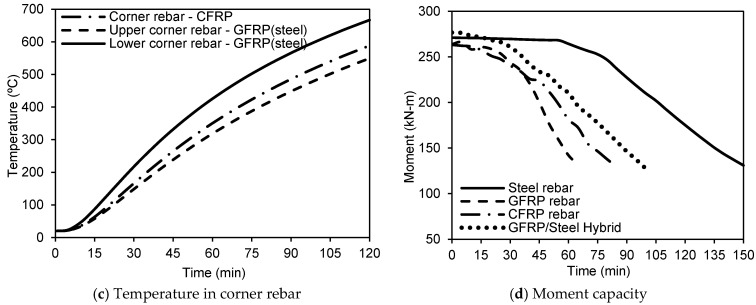
Typical response of concrete beam, with IR–FRP, under fire exposure. (**a**) Layout of beam. (**b**) Cross section of three beams. (**c**) Temperature rise in three beams. (**d**) Variation in moment capacity. Variation in temperature rise in steel and FRP rebars and beams’ cross section (as seen in [15]).

The response and extent of capacity degradation of a fire-exposed FRP–RC member depends not only on the type of FRP used but also on the type of application (IR, EB, or NSM) involved. To illustrate the effect of FRP type on the fire response of an IR FRP–RC member, three beams (designated as Beam III, Beam IV, and Beam V) were analyzed as part of a case study [15]. The layout of the beams, together with cross section details, is shown in Figure 4a. Beam III has eight *ϕ* 16 mm steel rebars (in two layers) as tensile reinforcement, while Beam IV has eight *ϕ* 16 mm GFRP rebars (in two layers), and Beam V has four *ϕ* 12.7 mm CFRP rebars (in one layer). These beams are designed to yield similar flexural capacity, and based on the room-temperature analysis, the nominal flexural capacities of Beam III, IV, and V are 271, 264, and 264 kN·m, respectively. Detailed information on beam characteristics and material properties, as well as analysis details, are presented in [15].

Figure 4c illustrates the thermal response characteristics of three beam configurations by comparing corner rebar temperature evolution as a function of fire exposure duration. GFRP reinforcement (Beam III) and steel reinforcement (Beam IV) exhibit comparable temperature progression profiles under fire exposure. The lower reinforcement layers in Beams III and IV demonstrate accelerated thermal response compared to upper layers, attributable to reduced cover thickness effects. However, GFRP reinforcement exhibits more pronounced degradation of mechanical properties at elevated temperatures relative to steel reinforcement. This results in diminished load-carrying capacity of Beam IV compared to Beam III at equivalent fire exposure durations. Beam V, incorporating single-layer CFRP reinforcement, demonstrates reduced thermal response rates due to increased concrete cover thickness. Additionally, CFRP’s superior thermal strength retention characteristics relative to GFRP result in enhanced strength throughout the duration of fire exposure.

Figure 4d depicts the temporal evolution of moment capacity for each beam configuration under fire exposure. The conventional RC beam (Beam III) maintains superior moment capacity compared to GFRP- and CFRP-reinforced configurations (Beams IV and V) throughout the exposure duration. This can be attributed to steel’s enhanced stability of mechanical properties. Specifically, Beam III maintains uncompromised moment capacity for 60 min of fire exposure, corresponding to steel’s thermal stability below 350 °C. Conversely, Beams IV and V exhibit immediate degradation of moment capacity upon fire exposure initiation. After 30 min of exposure, both configurations experience severe capacity reduction coinciding with polymer matrix decomposition (approximately 200 °C). GFRP-reinforced Beam IV demonstrates accelerated moment capacity degradation due to more rapid strength deterioration characteristics. Ultimate failure through flexural strength exceedance occurs at 150, 70, and 85 min for Beams III, IV, and V, respectively. This demonstrates conventional steel-reinforced concrete’s superior fire resistance compared to FRP-reinforced configurations, with CFRP-reinforced systems exhibiting moderately enhanced fire resistance compared to GFRP-reinforced configurations.

The fire response of the EB and NSM FRP incorporated beam follows a similar trend to that of an IR–FRP beam, as shown above. However, the fire resistance (or failure time) of an EB FRP-strengthened beam is lower than that of an NSM–FRP RC beam, which in turn is lower than that of an IR–FRP RC beam [16]. It is worth noting that a comparison related to fire resistance performance for different reinforcement methods (IR, EB, and NSM) can be found in a companion work [16]. It should also be noted that provision of appropriate insulation is key to minimizing the fire performance problems observed in fire tests [16].

## 4. Methods for Evaluating Fire Resistance

There are two broad methods for evaluating structural members’ fire resistance: prescriptive-based and rational engineering approaches. As mentioned earlier, prescriptive methods for evaluating fire resistance are derived from experience with fire testing. Rational methods involve the application of calculation methodologies for evaluating fire resistance. However, currently, there are very limited rational engineering approaches for the fire resistance evaluation of FRP–RC members. So, undertaking fire resistance tests is key to enabling the fire resistance information on FRP–RC members, both for deriving fire ratings and validating numerical models.

### 4.1. Standardized Test Methods for Evaluating Fire Resistance

The most conventional approach for assessing the fire resistance of building elements is through standardized fire resistance tests. Standard fire tests are conducted as per the testing procedures laid out in fire test standards adopted in each country, such as ASTM E119 (USA), UL 263 (USA), or ISO 834 (Europe and most other countries) standards [17,18,19]. For the test results to be accepted by the regulatory community, the specimen type, conditioning, test procedure (such as fire exposure, loading, etc.), and endpoint (failure) criteria must strictly follow the specifications laid out in these test standards.

The primary objective of these fire resistance evaluations is to establish quantified ratings for structural elements intended for building construction applications. The experimental data derived from these assessments serves to generate comparative fire resistance classifications across diverse structural member and building component typologies, which are subsequently compiled into standardized directories (exemplified by the UL directory) and design specifications for practical implementation. The fire resistance rating assignment methodology involves determining time-to-failure metrics, which are then conservatively rounded down to 30 min increments for durations up to 2 h, transitioning to hourly intervals thereafter.

### 4.2. Testing Procedure

Standard fire tests are conducted in specially designed fire-testing furnaces with specific features and dimensions. During the test, the structural member or assembly must be subjected to a standard fire exposure and structural load. The specified standard fire exposure is according to a time-temperature curve, in which the temperature increases for the entire test duration. The load applied on the test member is usually a service load (equivalent to dead plus live load). The standard fire curve to be followed as per ASTM E119, ISO 834, or ASTM E-1529 standards is shown in Figure 4. While ASTM E119 and ISO 834 fire curves are to be followed for evaluating the fire resistance of building elements, the ASTM E-1529 fire curve is for evaluating the fire resistance of structural members under hydrocarbon fire exposure. The specified fire exposure in other standards (followed worldwide) is quite similar but has minor variations.

Fire resistance of tested assemblies and members is quantified through their capacity to maintain structural and functional integrity under standardized fire exposure conditions. The metric is temporally defined, measuring the duration for which a structural element, component, or assembly sustains prescribed performance parameters. Standard fire resistance evaluation protocols incorporate three fundamental performance criteria: thermal *insulation* requirements governing temperature differential limitations and fire propagation control, structural *stability* parameters ensuring collapse prevention through maintenance of load-bearing capacity, and system *integrity* specifications controlling flame and smoke transmission. To achieve compliance, these criteria must be simultaneously satisfied throughout the designated fire resistance period.

### 4.3. Drawbacks and Limitations of Standard Fire Resistance Tests

Despite providing valuable data for fire resistance classification, standard fire testing protocols can exhibit significant methodological limitations. The test conditions fail to accurately replicate authentic fire loading scenarios and structural boundary conditions characteristic of actual building fire events. Fire resistance ratings derived from standardized testing may also yield excessively conservative specifications. A critical methodological constraint lies in the limitations of data acquisition protocols, which typically terminate at predetermined endpoint criteria (corresponding to target rating periods) without capturing post-rating behavioral characteristics of the structural element. These methodological constraints are comprehensively analyzed in the work of Kodur and Fike [20].

More importantly, the specifications in these fire test standards take into account most of the scenarios applicable to structural members made of concrete, steel, wood, masonry, etc. However, the specified test specifications cannot fully capture the fire behavior of FRP-incorporated structures since the presence of FRP leads to various changes in the member response, as illustrated in Section 3. As FRP is a recently introduced material in construction applications, these specifications do not cover certain performance issues specific to FRP-incorporated structural members. These specific factors are to be duly considered in fire tests to evaluate the realistic fire performance of FRP-incorporated structural members. These factors, which are specific to FRP-incorporated members, are laid out in Section 5.

## 5. Additional Protocols for Fire Testing of FRP–RC Structures

The most common approach for evaluating the fire resistance of structural members, as discussed above, is through standardized fire resistance tests. The procedure for undertaking such tests is specified in ASTM E119 [17], UL 263 [18], or ISO 834 [19] standards. The specifications in these test standards consider most scenarios applicable to structural members made of conventional materials such as concrete, steel, wood, and masonry. However, certain performance issues that are typical of FRP-incorporated structural members are not specifically covered in the specifications of ASTM E119. These additional factors must be duly considered in fire testing to evaluate the realistic fire performance of FRP-incorporated structural members. The additional testing protocols, to be adopted in fire testing of IR FRP-incorporated members, are discussed below. All other testing procedures, as laid out in ASTM E119, must be strictly followed to generate fire resistance ratings for use in practice.

**Specimen preparation:** To undertake a standard fire resistance test, a structural member with FRP reinforcement must be designed and fabricated per applicable specifications in concrete and FRP design codes [ex: ACI 318 and ACI 440 in the USA]. The concrete specimens are to be fully cured before applying any fire insulation. Also, following fire insulation application, the member must be sufficiently cured prior to undertaking a fire resistance test to meet the ASTM E119 (stated below) requirements regarding the moisture condition [17].

“All test specimens must be conditioned to attain a moisture content comparable to that in the field prior to testing. For uniformity, the standard moisture content is defined as that in equilibrium with an atmosphere of 50% relative humidity at 73 °F (23 °C). Massive concrete units requiring unusually long drying periods may be fire resistance tested after a 12-month conditioning period”.

As stated above, reaching the above-recommended moisture content levels in concrete (especially FRP–RC members) takes a long time. This factor must be considered when planning fire resistance tests since high moisture levels present in test specimens at the time of testing can be detrimental to fire resistance in an FRP-incorporated concrete member. Although ASTM lays out how the test result (fire resistance time) should be corrected to account for any variation from the standard moisture condition, it is preferable to test the specimen after reaching the recommended moisture levels. Further, any recommendations specified by the FRP and resin manufacturers regarding the right temperature and humidity levels should be considered.

**Figure 5:**
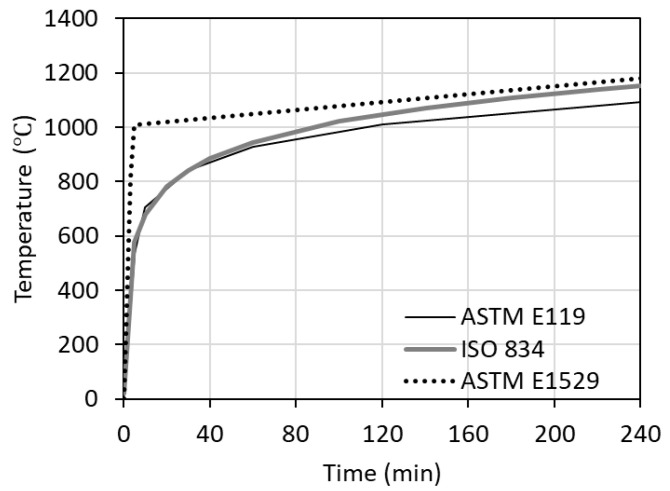
Fire exposure scenarios specified in fire test standards for undertaking fire resistance tests [17,18,19].

**Variation in fire exposure scenario:** It is worth noting that the ASTM E119 (or ISO 834, or ASTM E-1529) standard states that the furnace temperature must follow the time–temperature profile of the standard fire (as shown in Figure 5). Thus, the most important condition of a successful fire test is to ensure that measured temperature–time points in the furnace follow closely those of specified temperature–time points in the standard fire curve. However, these standards give some leeway for situations where the specified time–temperature profile during the fire test cannot be maintained fully. This factor can be taken advantage of while testing an FRP-incorporated structural member.

It is often hard to follow this condition in an FRP-incorporated structural member as FRPs can add fuel to the fire, and as a result, the temperature in the furnace can vary slightly, and it will take a bit of time to bring back the fire temperature to follow the standard fire curve. In such scenarios, ASTM E119 allows for an adjustment to the measured fire resistance with certain conditions, thus making the fire test results still useful [17]. Accordingly, if the maximum discrepancy of the area under the measured time–temperature curve is within 10% of the standard (E119) time–temperature curve for the first hour or it is within 7.5% for the next hour (i.e., up to 2 h) or within 5% after 2 h, the measured fire resistance period needs to be adjusted through a correction factor, C.

The correction factor, C, is to be calculated by taking into account the difference between measured and specified temperature–time curves in a fire test [21] and is given as

(1) $$C=2I(A-A_{\mathrm{st}})/3(A_{\mathrm{st}\;}+{\;L}_{g})$$

where

*C* = correction in the same units as *I*,
*I* = indicated fire-resistance period in the test,
*A* = area under the curve of indicated average furnace temperature for the first three-fourths of the indicated period,
*A_st_* = area under the standard furnace curve for the same part of the indicated period and
*L_g_* = lag correction in the same units as *A* and *A_s_* (30 °C·h (1800 °C·min)).

Knowing C, the actual fire resistance (FR_actual_) of a tested member can be evaluated as:

FR_actual_ = FR_measured_ ± C (2)

**Figure 6:**
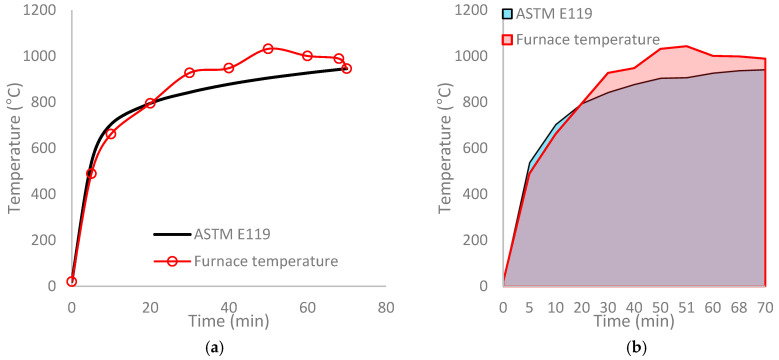
Comparison between the measured furnace temperature against the standard fire during a fire test. (**a**) Time–temperature data; (**b**) calculated areas under time–temperature curves [19].

Figure 6 illustrates the scenario during a fire test when such a correction factor can be effectively applied to correct the measured fire resistance in tests. The two curves in Figure 6a correspond to the standard furnace curve to be followed as per ASTM E119 and the average of the actual furnace temperatures measured during the fire test. Figure 6b illustrates the two areas (A_1_ and A_2_) under the time–temperature curves that can be used as inputs into Equation (1). The application of this correction factor to adjust the measured fire resistance is illustrated in Ref. [21].

**Load-level:** The load applied on the structural member during a fire test significantly influences the resulting fire resistance. ASTM E119 specifies that the applied loading on a structural member during fire exposure should equal maximum service loads (this translates to the sum of dead and live loads). This is approximately 50% of the ultimate capacity of the member for room temperature. For EB and NSM FRP–RC members, this capacity is based on the strengthened RC member, calculated according to applicable specifications for FRP–RC members (ex: ACI 440 [22]).

Recent provisions in ASCE-07 standard [23] state that the leading on a structural member in a fire situation should be estimated based on 1.2DL + 0.5LL. Other international standards also specify reduced load levels during fire exposure, and this is due to the fact that fire is considered a rare event in design (See Table 1). This new provision, i.e., reduced load level, is often beneficial from the point of fire resistance and can be adopted in undertaking fire resistance tests and calculation methods (as long as the applied load level on the member is documented in the test report).

**Instrumentation:** ASTM E119 [17] and other fire test standards list requirements for mounting instrumentation on the test specimen. This typically includes thermocouples and deflection gauges to monitor the temperatures. In the case of conventional concrete members (with metallic reinforcement), these thermocouples are placed on the tensile steel rebars at the critical section and on the unexposed surface in the case of floors and walls. However, this may not be sufficient to capture the full response of the member during fire exposure in the case of FRP–RC members.

**Figure 7:**
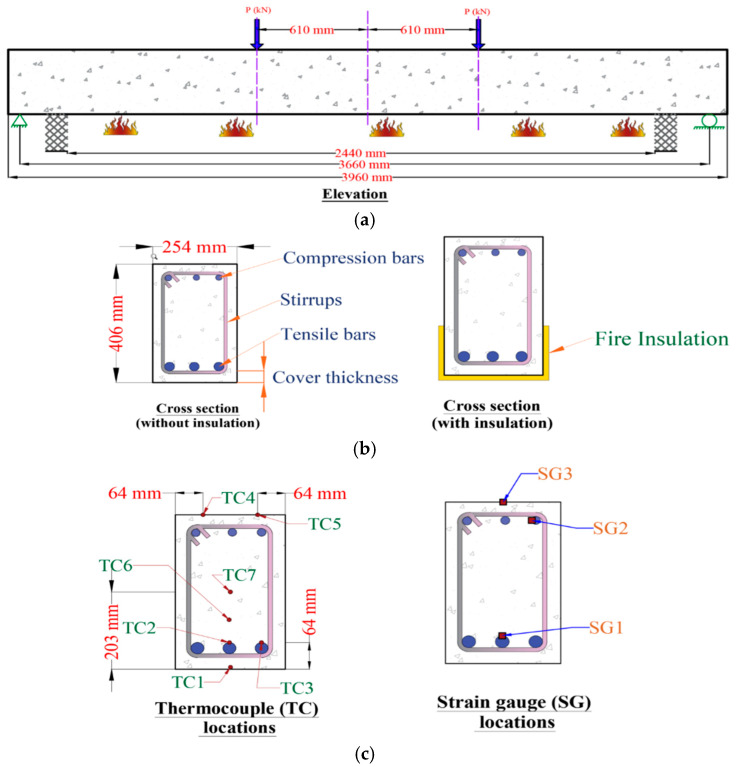
Proposed instrumentation details at the critical section for undertaking fire tests on FRP–RC beams: (**a**) beam layout (elevation); (**b**) cross section; (with and without insulation); (**c**) thermocouple and strain gauge locations.

Additional thermocouples need to be placed at the critical sections of the cross section, including on the FRP reinforcement, concrete, and on the insulation (if present) at the critical section. Additionally, thermocouples need to be placed on FRP in the anchorage zones to monitor any debonding or delamination of FRP under high-temperature effects. This extra temperature data are useful for evaluating the time when T_g_ in FRP is reached and also the anchorage zone’s effectiveness, helping further analyze the members’ fire performance. The critical sections for placing the thermocouples and strain gauges in an FRP-reinforced beam are shown in Figure 7.

In addition to thermocouples, strain gauges can be installed on FRP reinforcement (specifically in IR and NSM applications) to collect strain data and analyze the strength contribution of FRP during fire exposure. Although conventional strain gauges may not provide reliable strain data beyond 100 C, high-temperature strain gauges can provide reliable data up to 400 °C. Due to the possibility of some of the thermocouples (and strain gauges) malfunctioning at extreme temperatures, it is recommended that additional backup thermocouples be installed at strategic locations. This extra data can help learn from the fire tests and develop strategies for improved fire performance of FRP–RC members. Visual observations should also be made during the fire test through available window ports in the furnace. These observations can provide deeper insights into the fire performance of FRP–RC members.

**Coupon tests:** As part of fire tests, a set of strength tests are to be carried out on concrete and FRP coupons to determine the actual strength of concrete and FRP. This is because the actual strength of FRP (and concrete on the test day) often significantly varies from the manufacturer’s specified (design) strength and this can affect the fire performance. For this purpose, concrete cylinders and FRP coupons must be prepared to undertake material characterization tests to evaluate their strength properties at the time of fabrication. The casted concrete cylinders, just like fire test specimens, must be cured under ambient temperature for 28 days, stored at 25 °C and 40% relative humidity, and then prepared for undertaking strength tests. The strength tests of concrete cylinders will have to be carried out to evaluate the compressive strength at 28 days and on the fire test on FRP–RC members. Similarly, tensile strength tests on FRP coupons have to be carried out, as per the procedures laid out in the applicable test standards, to evaluate the tensile strength of FRP.

**Test observations:** FRP is a combustible material (unlike concrete and steel), and the changes that occur in FRP during fire exposure play a critical role in the fire resistance of FRP-incorporated RC members. For this reason, key test observations and thermocouple and deflection data recommended in ASTM E119 are to be recorded. These test observations can shed light on drawing inferences about the fire performance of the FRP–RC member. The visual observations at regular intervals should be taken through window ports in the furnace to gauge the condition of FRP (such as flaming, decomposition, debonding, and delamination) and that of insulation (cracking and delamination). These observations can provide useful lessons for designing future test specimens and selecting appropriate materials for the structural system.

**Failure criteria:** ASTM E119 specifies three failure criteria that are to be satisfied in standard fire resistance test on a structural member, namely the *insulation* (or barrier) criterion to limit the temperature rise and fire propagation, the *stability* (or strength) criterion to prevent collapse, and the *integrity* criterion to limit fire (flame or smoke) spread. When it comes to FRP-incorporated members, there is a misconception among the wider community that the failure corresponds to reaching T_g_ in FRP. Reported test data clearly infer reaching glass transition temperature (T_g_) in FRP during a fire event does not lead to the failure of an FRP-incorporated concrete member [5,6,10,11]. Further, experience shows that structural failure follows when the thermal (such as steel or FRP rebar reaching critical temperature, T_c_) or deflection limit state is first reached. Therefore, the fire test may continue beyond the thermal and deflection limit states to reach the strength failure limit state, but only if it is deemed safe during the fire test. In fact, an FRP-reinforced concrete member can continue to carry the load much beyond T_g_’s reach. For this reason, the failure of the FRP–RC member should be based on the strength (capacity) of the member, when it is no longer adequate to carry the expected loads during a fire, and not just at the point when the FRP reaches its T_g_ or steel rebars attain T_c_.

## 6. Conclusions

Fiber-reinforced polymers (FRPs) have emerged as a promising material for reinforcement in concrete structures, particularly for enhancing the durability and performance of concrete structures. The fire response of FRP-incorporated concrete structures is altered due to the presence of FRP. However, the fire resistance of these FRP-incorporated structural members continues to be evaluated through standard fire resistance test procedures as applied to conventional steel and concrete structural members. By following this approach, certain performance issues typical of FRP-incorporated structural members cannot be captured when evaluating the fire performance of FRP-incorporated RC members, accounting for specifically covered in the specifications of ASTM E119. The contradictions concerning the fire performance of FRP–RC structures are highlighted, and the drawbacks in the current fire testing approach that make them not fully applicable to FRP–RC structures are discussed.

A set of additional testing protocols and procedures for undertaking fire resistance tests on FRP-incorporated concrete structural members are proposed. The performance criteria to be applied for evaluating the failure of FRP–RC structural members under fire conditions are also presented. These protocols are envisioned to advance the current knowledge gap and practical limitations associated with using FRPs under fire conditions. These are also likely to complement and update some of the current codal provisions present in building codes and standards. Finally, the state of the art, with regard to fire testing of FRPs, can be improved by examining their response under realistic fire conditions and boundary conditions. Such examination may lead to generating new protocols to explore the use of anchoraged insulations or continuous elements.

## Figures and Tables

**Table 1:** Load combinations for structural fire design in different codes and standards.

Code	Load Combination	Remarks
ASTM E119	*U = D + L*	Mostly used for evaluating fire resistance of members through perspective-based approaches
ASCE 7	*U* = (1.2 or 0.9) *D* + *A_k_* + 0.5 *L +* 0.2*S*	If *D* has a stabilizing effect, then this factor of 1.2 may be reduced to 0.9. *A_k_* = load or load effect (such as fire-induced restraint) resulting from extraordinary event *A* (fire).
Eurocode 1	*U* = 1.0 *D* + *P L*	Load combination can vary slightly depending on whether the fire load is taken to be of a frequent or quasi-permanent nature. *P* = 0.5 for residential and office buildings 0.7 for shopping areas, and 0.9 for storage areas.
British Code	*U = D + L* + 0.33*W*	Wind loads should only be applied to building members where the height to eaves is greater than 8 m and are only considered when checking the design of the primary elements of the framework.
Australia/New Zealand Standard	*U* = 1.0 *D* + *M L*	*M* = 0.4 for residential and office buildings and 0.6 for occupancies with semi-permanent live loads.

*U* = factored loads specified in common fire codes, *D* = dead load, and *L* = live load.

## Data Availability

Some or all data, models, or code that support the findings of this study are available from the corresponding author upon reasonable request.
